# Validation of PleaseApp: a digital tool for the assessment of receptive pragmatic abilities in children with neurodevelopmental disorders

**DOI:** 10.3389/fpsyt.2024.1329022

**Published:** 2024-01-26

**Authors:** Clara Andrés-Roqueta, Raquel Flores-Buils, Alfonso Igualada

**Affiliations:** ^1^Department of Developmental, Educational Social and Methodological Psychology, Universitat Jaume I, Castellón de la Plana, Spain; ^2^Faculty of Psychology and Education Sciences, Universitat Oberta de Catalunya, Barcelona, Spain; ^3^Institut Guttmann, Institut Universitari de Neurorehabilitació, Barcelona, Spain

**Keywords:** assessment, digital tool, psychometric properties, pragmatics, social communication, neurodevelopmental disorders, children

## Abstract

**Background:**

Pragmatic skills allow children to use language for social purposes, that is, to communicate and interact with people. Most children with neurodevelopmental disorders (NDDs) face pragmatic difficulties during development. Nevertheless, pragmatic skills are often only partially assessed because the existing instruments usually focus on specific aspects of pragmatics and are not always adapted to children with communication difficulties. In this sense, digital tools (e.g., apps) are an optimal method to compensate for some difficulties. Moreover, there is a lack of pragmatic tools measuring the receptive domain. Therefore, the present study aims to validate PleaseApp as a digital instrument that measures eight pragmatic skills by presenting the design of the assessment tool and its psychometric properties.

**Methods:**

PleaseApp was designed based on previous empirical studies of developmental pragmatics in children with and without NDD. PleaseApp assesses eight receptive pragmatic skills: figurative language, narrative, reference, indirect speech acts, visual and verbal humor, gesture-speech integration, politeness, and complex intentionality. The study involved 150 typically developing children between 5 and 12 years of age.

**Results:**

A confirmatory factor analysis proposes an eight-factor model with no underlying factor structure. The eight tests that compose PleaseApp have obtained a model with a good fit and with adequate reliability and validity indices.

**Discussion:**

PleaseApp is an objective, valid, and reliable tool for assessing pragmatic skills in children with NDD. In this sense, it helps to assess whether a child has acquired pragmatic skills correctly according to his/her age and clarify the specific problems a child has in eight different components to plan personal and personalized interventions.

## 1 Introduction

### 1.1 Pragmatics

Language is made up of phonetic-phonological, lexical-semantic, morphosyntactic, and pragmatic skills. Pragmatic skills allow us to appropriately use language in context or a communicative situation (1). In this sense, information from the context includes physical aspects of the situation where the conversation occurs and the social, cognitive, and linguistic context of the discourse (2). Consequently, the acquisition and development of pragmatic skills depend on linguistic abilities (e.g., structural language and exposure to conversations) (3, 4) and theory of mind skills to correctly infer the communicative intention of the speaker during a dialog (5).

Importantly, pragmatic competence is a multidimensional phenomenon that encompasses a wide range of interdependent expressive and receptive skills that are linked to other developmental skills to a greater or lesser degree. Therefore, its acquisition and development also cover a wide range of ages throughout childhood and adolescence (6, 7). It should be noted that the subcomponents of pragmatics may be variable depending on the literature or the field of knowledge consulted since pragmatics is a multidisciplinary area in which studies that come from psychology, speech-language therapy, and linguistics come together.

In this sense, there is a need for multidimensional approaches to pragmatic development to create a receptive method of assessment and to apply it in the health and education fields. Receptive pragmatic assessments would allow us to assess children’s pragmatic skills as listeners, which is important to ensure success in conversation. Some of the most relevant pragmatic receptive skills are the following:

- *Figurative language* understanding. This ability involves deducing when the speaker’s productions have a different meaning (figurative) than what is actually expressed (literal) (4, 8). For its understanding, it is necessary to inhibit literal meaning and include the comprehension of metaphors (novel and conventional), idioms, or similes, among others (9, 10). In this sense, similes are considered easier than idioms or metaphors to understand because similes contain an explicit syntactic cue (e.g., “like”) that a comparison is necessary (8).
- *Narrative*. Narrative skills include mainly expressive abilities to generate a story and to retell it after having heard it (11). However, before generating a story (telling or retelling), children must use their receptive pragmatic skills to order the given episodes (e.g., pictures) using details of contextual information (mainly cognitive, social, and linguistic details) to construct a coherent and chronologically ordered story (2).
- *Reference.* Reference skills allow a speaker to describe or represent reality through language, providing enough information so that another person can understand it (12). In this sense, the listener also has expectations about the optimality of the reference expressions used by the speaker, and he must detect when these are not met (e.g., when the speaker does not provide enough information or it is ambiguous). Therefore, he must request more information or clarification (13).
- *Indirect speech acts.* Speech acts can be classified as direct and indirect acts (14). Indirect acts are used to communicate more information than what is actually said (e.g., using insinuations). Thus, to understand indirect speech acts (that is, to understand the actual intentionality of a speaker), a listener must grasp aspects linked to the theory of mind, especially in relation to the recognition of facial expressions or intonation (15).
- *Humor*. Understanding humor requires making inferences to the context and the communicative intentions of the speaker, to understand the ludic or funny meaning (16). Humor occurs when there is a discrepancy between what is expected and what really happens or is perceived (17). The incongruity and resolution that leads to finding humor can occur both through a visual element or in a verbal element of the context (e.g., pictures or sentences).
- *Gesture-speech integration*. Multimodal skills include the ability to integrate iconic gestures with speech to improve the understanding of words and messages, especially if it is complex. In this sense, a listener must be able to integrate gestures (to complement information, supplement information, or finish a sentence) and the sentences of a speaker in a conversation (18, 19).
- *Politeness*. Courtesy consists of being polite to others or showing solidarity and kindness. It requires adapting linguistic behavior by choosing the appropriate words and understanding the mental states of people and the social norms of the situation (20). In addition, it requires a social understanding of interpersonal relationships, such as relationships with the speaker or vertical relationships (21).
- *Complex intentionality.* It includes the ability to understand the communicative speaker’s intention hidden behind a non-literal, indirect, or false message (22). A speaker breaks these pragmatic rules both deliberately (e.g., when lying or being ironic to make a joke) as well as non-deliberate (e.g., when committing a mistake or confusion). In this sense, it is considered a metapragmatic skill (20), and theory of mind skills are essential to infer the actual intentions and other mental states of the people involved (9, 23).

Pragmatic skills are a key aspect of socialization with peers in inclusive settings, and therefore, accurately detecting what specific problems a child has in these areas would allow these skills to be improved and treated in a way adapted to real needs (24).

### 1.2 Typical and atypical pragmatic development

For most children, the ability to use language to communicate with others is a taken-for-granted skill. However, most children with neurodevelopmental disorders (NDDs) present pragmatic difficulties of a greater or lesser extent as a consequence of the implication of linguistic and cognitive factors in the correct development of pragmatic skills (2), which prevents them from taking part in daily social activities (e.g., at school or family environments). It is important to note that children must have developed sufficient structural language skills before higher-order pragmatic deficits can be detected (5–6 years), but difficulties may be latent during the preschool period (24, 25).

Among some of the child NDD populations at greatest risk of suffering difficulties in the acquisition and development of pragmatic skills is the *Autism Spectrum Disorder (ASD).* Regarding *figurative language*, various empirical and theoretical studies describe general difficulties in this area (e.g., 8, 9, 26), as well as specific difficulties in novel metaphors (27) or idioms (4). In this sense, impairments on the metaphor tasks seemed to be linked to language impairment within the disorder regardless of autistic features (8). Regarding *narrative skills* related to ordering a story from pictures, autistic children do not have special difficulty in ordering causal or mechanical scenarios or referring to everyday routines, but difficulties appear when ordering episodes, which include the mental and psychological states of the people (28). Similarly, they have difficulties in narrative production and when it comes to realizing inferences in narratives, including issues with coherence, connection between events, and/or giving irrelevant information or saying unusual or bizarre comments, among others (2, 29, 30). In relation to *reference skills*, autistic children manifest both expressive problems and receptive problems, such as detecting violations of conversational maxims related to quantity (e.g., make your contribution as informative as required) (31, 32). Regarding *indirect speech acts* comprehension, various studies describe both difficulties and strengths in autistic people, and results are often mixed in most cases depending on their level of structural language and their age. For example, difficulties in understanding indirect requests in children and autistic adults have been demonstrated (30, 33), but some strengths are found in autistic adults (34), preadolescents (35), and children (36) with a better level of language. In relation to *humor* understanding, studies demonstrate the existence of problems understanding some forms of humor from childhood to adolescence (17, 30). Specifically, autistic people can understand certain types of humor (from puns, antics, or simple jokes to very clever and precisely formulated comments), but they have more difficulty solving mentalistic-type jokes (37). Moreover, the veracity of the context can influence their sense of humor since their creativity is based more on reality than on imagination or fiction. Regarding *gesture-speech integration*, a low competence has been demonstrated as well (38). Regarding *politeness*, there are some studies that describe the difficulties these children have in using some forms of courtesy (39). Finally, in relation to *complex intentionality*, studies show both difficulty in understanding mistakes, that is, discerning intentionality from unintentionality (23, 40), and also correctly understanding masked communicative intentions (9, 31, 41). Similarly, the difficulty in understanding irony has also been detailed in a more concrete way, closely related to their theory of mind skills (34).

Moreover, regarding children with *Communication Disorders,* various studies have observed difficulties in pragmatic components in both children with Social Communication Disorder (SCD) and children with Developmental Language Impairment (DLD), although they are usually less pronounced in children with DLD (29). Regarding *Figurative language*, these difficulties have been described both for SCD and DLD in understanding idioms (42, 43) and for DLD in novel metaphors (8). Moreover, most studies have focused on children with DLD (e.g., as a control group for autistic children or to better study the role of structural language in pragmatic difficulties). In this sense, some studies describe that children with DLD have difficulties in *narrative* production or inferring information from narratives (2, 44) and in understanding *indirect requests* (45). Difficulties in *reference skills* have also been observed in children with DLD (13), as well as identifying uninformative quantifiers (46) or detecting violations of conversational maxims related to informativeness (31). Regarding *humor*, some studies describe difficulties in understanding graphic humor for children (47) and general humor in the adolescent population (48). Similarly, some studies also describe difficulties in *gesture-speech integration* (18, 49). In relation to *politeness*, certain difficulties in the use of politeness formulas have been stated (32). Finally, regarding c*omplex intentionality*, different studies show difficulties in understanding mistakes and faux pas (50) or irony (31) for children with DLD, as well as difficulties in understanding masked communicative intentions for both DLD and SCD (47, 51).

To a lesser extent, children with *Attention Deficit Hyperactivity Disorder (ADHD)* also present more pragmatic difficulties than their peers with typical development, although not with the same severity as autistic children (52). Regarding *Figurative language*, they have difficulties understanding figurative language with and without context, as well as idioms (53). In relation to *narrative* skills, some studies describe difficulties in narrative production (e.g., topic maintenance, event sequencing, and referencing), which are evident over and above general language functioning (54). Regarding *reference*, again, the studies found are fewer, but some difficulty is also demonstrated in their reference skills (55). Similarly, they have difficulty understanding some *indirect requests* (53). Regarding *humor,* some studies have found that it is an area of strength in these people since low inhibitory control is advantageous for divergent thinking (56). In this sense, difficulties in the appreciation of humor have only been demonstrated in children with ADHD with comorbidity with non-verbal learning disorder (57). Regarding *gesture-speech integration*, no explicit evidence has been found, although some difficulty in the perception of non-verbal cues has been demonstrated. Moreover, some difficulty is described in some forms of *politeness* (58). Finally, regarding *complex intentionality*, there is evidence that their primary difficulties in executive function can lead to difficulties in understanding mistakes, irony, and intentionality (59). It must be noted that these difficulties are related to other pragmatic expressive components such as the management of social discourse (impulsive speech, interruption of conversations and inappropriate initiations, loss of information in the dialog, and little attention paid to context) (54). In fact, there are studies that have shown that these partially explain the high rates of social incompetence (54).

Finally, it is important to note that children with *Intellectual Disabilities*, as a consequence of general cognitive difficulties, also show pragmatic difficulties such as understanding long and complex conversations, understanding deceptions, double meanings, and metaphors, or organization of discourse. Specifically, the existence of specific pragmatic difficulties has been studied in Williams Syndrome, Fragile X syndrome (60), and Down syndrome (61).

This bulk of evidence shows that different pragmatic abilities are relevant to children’s development and differentiate profiles in children with pragmatic needs, which motivates the assessment of these abilities. Moreover, the population with NDD has shown a great variability of pragmatic abilities with potential difficulties in one or more of its subcomponents, which requires a comprehensive assessment to identify the strengths and weaknesses of the child’s pragmatic skills (25, 62).

### 1.3 Pragmatic assessment

Pragmatic assessment is one of the linguistic components that has received less attention in clinical research. However, there are some assessment tools measuring pragmatic abilities with different methodologies. In this sense, pragmatic skills are usually assessed through questionnaires filled out by parents or teachers (e.g., Children’s Communication Checklist-2) (63) and through observation measures by professionals (e.g., the pragmatic component of Clinical Evaluation of Language Fundamentals–Fifth Edition or CELF-5) (64). In this sense, some of the existing tests are designed in other countries (generally English-speaking), and some items are not valid for other cultures (65). Regarding the methods used by experimental pragmatics research, these investigations have developed a wide set of empirically validated and research-based methods that extract direct measures of comprehensive pragmatic abilities. The design of these tasks has isolated different pragmatic capabilities in the comprehensive domain by quantifying the number of correct responses. However, to the best of our knowledge, there is a lack of pragmatic assessment tools with direct measures of pragmatic comprehensive skills that implement evidence-based methodologies.

On the other hand, it is difficult to find assessment tools that cover the full set of existing pragmatic skills in the expressive and comprehensive domains and that give a comprehensive view of pragmatic ability across the entire developmental age range (24, 25). Moreover, existing pragmatic tools tend to focus on specific aspects of pragmatics (e.g., understanding of figurative language or use of conversational skills) and forget certain essential aspects (e.g., theory of mind skills or dimensions empirically studied by leading authors like reference skills) (3). To the best of our knowledge, there is a lack of formal assessment tools that integrate different measurements of pragmatic receptive abilities, which would better inform the actual competences of children with NDD in the comprehensive domain.

### 1.4 Digital assessment tools

In recent years, technology-based assessments have been increasing, as they provide a motivating and attractive environment for children (with and without NDD) and they prefer them to more traditional methods (66). Moreover, additional processing time and reduced anxiety were associated with face-to-face interactions for people with NDD, such as autistic children (67). Regarding the assessment of pragmatic abilities, digital formats also provide innovative ways to provide information about contextual factors that are crucial for the assessment of pragmatic disorders (25). Thus, in the pragmatic area, technology-based assessments offer a unique opportunity to create communicative contexts as similar as possible to real communicative situations, as they allow multimodality, such as the use of audio, image, movement, and text (e.g., audio recordings to provide structural language information, background images of the context to be integrated with the information, or the interaction with response buttons that include contextual information). Nevertheless, they cannot substitute real communicative contexts and real interactions with people, so the information that they provide must be used together or matched with other pragmatic ecological assessments if possible (e.g., observational measures or questionnaires) to have a better assessment of the real pragmatic behaviors of children.

Digital tools also have great potentialities in health, as well as in other disciplines, as they can be used both in face-to-face and teleintervention formats. Even though assessment tools to diagnose NDD have typically been created in an analogic format, research has already explored its online use with teleassessment practices (e.g., 68). In fact, the COVID-19 pandemic served to develop remote diagnosis and intervention models for autistic people (69). In line with this, professionals used assessment strategies during the COVID-19 pandemic lockdowns as an alternative method of service delivery (70). Moreover, teleintervention has already been shown to be a valid and effective modality in the screening and diagnosis of children with socio-communicative needs (71). In general, telehealth facilitates the diagnosis of children with developmental concerns (72). However, these studies demonstrate the feasibility, effectiveness, and diagnostic accuracy of teleassessment tools and protocols that have been adapted to a digital format. In this sense, there is a need for innovative digital assessment tools that integrate evidence-based procedures newly created in an online format that can be administered in face-to-face and online formats.

### 1.5 Aim of the study

For this reason, the design of PleaseApp was considered to provide the clinical and scientific community with a formal measure that allows the evaluation of receptive pragmatic skills. PleaseApp aims to expand the number of pragmatics components (and items) to carry out a complete assessment of pragmatics that contributes to making a differential diagnosis between those disorders that present comorbidity in this area. In this sense, item variations of PleaseApp (e.g., including the theory of mind content in some items) have the potential to be evaluated through the tool that would help in some cases to determine a specific diagnosis toward pragmatic difficulties.

Moreover, PleaseApp will allow establishing differences between and within disorders, pointing out weaknesses and strengths in the different components and subcomponents of pragmatics, and also allow the type of error children make when they do not answer correctly. Finally, the variation of items and the inclusion of the theory of mind contents will allow the assessment of the theory of mind’s comorbid difficulties.

Therefore, the aim of the present study is to evaluate the psychometric characteristics of the digital tool PleaseApp as a formal assessment of receptive pragmatics in a sample of primary school children and to analyze the data obtained, taking into account the age and sex of the participants.

## 2 Methods

### 2.1 Design of the tool

The development and design of PleaseApp include different steps. In the first step, previous scientific evidence about pragmatics and existing instruments were analyzed. On the one hand, several theoretical and empirical studies on the developmental milestones of pragmatic skills in typically developing children were reviewed, as well as the scientific procedures for the assessment and intervention of these skills. On the other hand, the specific strengths and difficulties common in children with different NDDs were also reviewed, such as ASD, DLD, SCD, or ADHD. This helped us to establish the structure and different subtests of the app and the variations between items within each level.

In the second step, 10 different levels were created, with 12 items per level. However, the data from the validation of the present study allowed us to define the items and levels that had adequate psychometric properties to be part of the test. So, the current version has only eight subtests because the subtests related to lexical inference from contextual information and metapragmatic skills in conversation were excluded due to the reduction of valid items.

Moreover, PleaseApp screening happens in eight scenarios familiar to the children (e.g., school) that contextualize each level and its plot. This is an attempt to make sense of the instruction given to the child at the beginning of each level, and to engage the child in the story requires employing his or her receptive pragmatic skills correctly.

Table 1 shows a definition of each subtest (level), and the variation of its items is presented. Moreover, references to the most relevant empirical or theoretical studies for the design and construction of each of the levels are also included.

**Table 1:** Definition of each subtest (skill and level) of PleaseApp and the variation of its items is presented.

Skill	Level task mechanism	Item variation	Example of the gradations of response (related to items presented in Figure 1)
Narrative (NARR)	*Cinema*. Put in order the different scenes of a movie.	Mechanical, behavioral, and mentalistic stories (28).	2 points: correct order of the four pictures (In the example: 3-4-1-2).
			1 point: order with correct ending (In the example, combinations different to the correct one but with number 2 as the last picture).
			0 points: other possible order combinations.
Politeness (POL)	*Train*. Choose the correct, polite expression and avoid those that are too direct (rude) or incoherent.	Greetings and goodbye, thanking, forgiveness, apologizing, asking for permission, and presenting or rejecting a gift through a white lie, among others (73–75).	2 points: polite and coherent (*Excuse me, can I seat here?*)
			1 point: rude and coherent (*Ei, I want to seat here*).
			0 points: polite but incoherent (*Excuse me, why are the seats green?*).
Reference (REF)	*Kitchen*. Ask (or not) for more information to find the correct object on the shelf because the cook sometimes is underinformative.	Scalar (large and small) and absolute (open and closed) implicatures. Multimodal elements (e.g., manual pointing gesture) (13).	Chef: “*Pass me the pineapple*.”
			2 points: Informative (First the child presses “?,” the chef specifies if the big or the small one, and then the child presses the correct referent).
			1 point: Underinformative (The child presses one of the pineapples, without pressing “?” before).
			0 points: Incorrect (Another combination, e.g., pressing the orange).
Indirect speech acts (IND)	*School*. Decide what to respond when non-explicit information is given to you.	Intentionality functions: directive, commissive, expressive, and assertive (15). Formats: statements and questions (33).	Boy: “*Ugh, how heavy is this backpack…”*
			2 points: the correct indirect speech act is inferred (Do you want me to bring you a book?).
			1 point: a different indirect speech act is inferred (You can buy another one with wheels).
			0 points: a literal meaning is inferred (I think it will weigh about 10 kilos).
Complex intentionality (INT)	*Beach*. Decide why characters are saying these sentences.	Intentionality/non-intentionality (e.g., faux pas, mistakes, confusions, errors versus lies or white lies) (9, 23), irony (sarcasm, questions, statements, exaggerations) (76), and the moral valence of the main intention of the speaker (positive or negative) (38).	Girl: “*Do you have any ice cream left?.”*
			Boy: “*No, I do not.*”
			Question: Why does he say “No, I do not.”
			2 points: the correct intention is inferred (*Because he does not want to give it to her*.).
			1 point: another intention is inferred (*Because he has forgotten he has it*.).
			0 points: response is based on literal or incoherent aspects (*Because he has already eaten it*).
Gesture and speech integration (GES)	*Circus*. Decide the correct meaning by integrating the information expressed in the gestural modality with the information expressed through speech.	Semantic function of the gesture: complementary, supplementary, replace part of the sentence. Grammatical category: actions and objects (18, 19).	Ballerina: “*While I was cutting things for the show*.”
			2 points: correct object (*scissors*).
			1 point: semantic competitor (*knife*).
			0 points: gesture competitor (*clothespin*).
Humor (HUM)	*TV*. Detect the inconsistency by selecting the visual or verbal element that makes the image funny.	Visual or verbal (17, 77). Type of humor (puns, semantic analogies, and mentalistic vignettes) (37). Type of protagonists in the scenes (people or fictional animals) (78).	2 points: funny and coherent (In the example, picture 3).
			1 point: not funny but coherent (In the example, picture 2).
			0 points: funny but not coherent (In the example, picture 1).
Figurative language (FIG)	*Zoo*. Choose the image that fits in the figurative meaning that the character is using.	Conventional metaphors (idioms), novel metaphors, and similes (4, 8–10). Variation of the topic (what it refers to) and the vehicle (what it is compared to) (10).	Zookeeper: The parrot house does not get the sun; it is like a fridge
			2 points: figurative meaning (In the example, picture 1).
			1 point: literal but likely meaning (In the example, picture 2).
			0 points: literal and unlikely meaning (In the example, picture 3).

**Figure 1:**
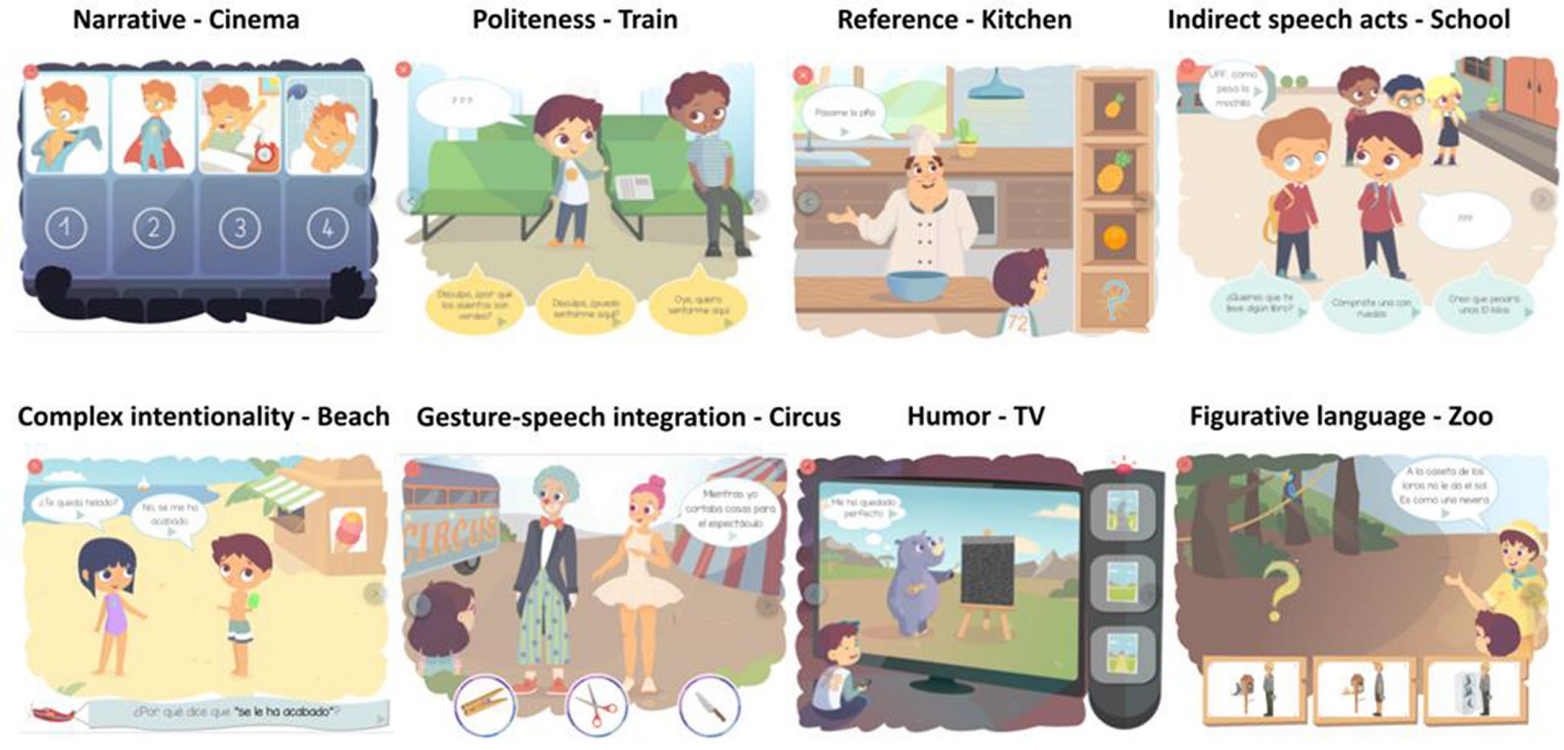
Examples of PleaseApp items.

Moreover, an example is given of the three gradations of response related to examples of items provided in Figure 1.

An example of an item from each of the levels is given in Figure 1.

Each item has three types of response coding: one is considered a correct response and the other two are considered incorrect. These three gradations of the response have been established depending on their use of pragmatic skills to go beyond the explicit or literal meaning, taking criteria of appropriateness (relevance of the answer given to the question that was asked), accuracy (informativeness), and veracity (truthfulness of the information provided), and following scorings similar to Andrés-Roqueta and Katsos (31). Therefore, each item is scored with 2, 1, or 0 points. For example, at the Figurative Language level, the three response options refer to figurative meaning (2 points), literal but probable meaning (1 point), and incoherent/inconsistent literal meaning (0 points).

### 2.2 Participants

A total of 153 boys and girls who attended different mainstream schools in the Valencian Community (Spain) were recruited in the present validation study, of which 54.9% were boys and 45.09% were girls. The distribution by age and sex is shown in Table 2.

**Table 2:** Age and sex descriptives of the sample.

Age	*n* (%)	Sex *n* (%)		
		Boys	Girls	Sex %
5 years	13 (8.49%)	7 (8.3%)	6 (8.7%)	53.84% / 46.15%
6 years	16 (10.45%)	10 (11.9%)	6 (8.7%)	62.5% / 37.5%
7 years	24 (15.68%)	13 (15.5%)	11 (15.9%)	54.16% / 45.83%
8 years	19 (12.41%)	10 (11.9%)	9 (13.0%)	52.63% / 47.36%
9 years	20 (13.07%)	10 (11.9%)	10 (14.5%)	50% / 50%
10 years	24 (15.68%)	15 (17.9%)	9 (13.0%)	62.5% / 37.5%
11 years	26 (16.99%)	14 (16.7%)	12 (17.4%)	53.84% / 46.15%
12 years	11 (7.18%)	5 (6.0%)	6 (8.7%)	45.45% / 54.54%
Total	153 (100%)	84 (100%)	69 (100%)	54.9% / 45.09%

### 2.3 Procedure

As discussed in the Method section on PleaseApp design, the authors designed a battery of 12 items for each of the 10 initial assessment levels following the theoretical review. Out of these 12 items, the first two items were test items, and they were used to ensure the child’s understanding of the instruction, and the next 10 items were the assessment test. Thus, the initial version of the instrument was composed of 120 items (20 test items and 100 assessment items).

After obtaining the authorization of the regional government, the ethics committee of the university, where the project was carried out, and the school authorities, informed consent was requested from the parents of the participating children.

First, this initial version of the instrument was administered to a pilot group of 16 children (two children of each of the target ages of the digital tool App, that is, from 5 to 12 years) to assess the comprehension and appropriateness of the items and instructions.

Then, the research group administered PleaseApp individually to each participant of the sample using computers, tablets, and laptops from the school and from the research groups, together with other standardized tests presented in the section on instruments.

Once the data were collected, the psychometric properties of PleaseApp were analyzed. For this purpose, construct validity analysis was performed by means of confirmatory factor analysis (CFA), reliability analysis, and convergent validity.

These analyses led to the elimination of a number of items due to their low correlation within their level and the elimination of two levels (metapragmatic in conversation and new lexical contextual inference) that did not obtain a model with adequate properties, thus configuring the final structure of the tool, which is presented in the Results section.

### 2.4 Instruments

#### 2.4.1 PleaseApp

PleaseApp (79) is a new digital assessment tool for receptive pragmatic abilities that includes eight tests of different pragmatic skills. In total, it consists of 77 items, 8 test items (1 at each level), and 69 assessment items scored with 2, 1, or 0 points. These items are distributed among eight levels as follows: narrative (NARR, cinema) 10 items, score range 0–20; politeness (POL, train) 10 items, score range 0–20; reference (REF, kitchen) 8 items, score range 0–16; gesture-speech integration (GES, circus) 8 items, score range 0–16; indirect speech acts (IND, school) 6 items, score range 0–12; complex intentionality (INT, beach) 8 items, score range 0–16; visual and verbal humor comprehension (HUM, TV) 8 items, score range 0–16; and figurative language (FIG, zoo) 11 items, score range 0–22. Its total score range is 0–138. It has good psychometric properties, which are presented in the present study.

#### 2.4.2 Pragmatic formal measure: BLOC-S-R

*The pragmatic subtest of the Objective Language Criteria Test–Screening Revised (Batería de lenguaje objetiva y criterial, BLOC-S-R)* (80) was used to have a measure of the expressive pragmatic competence of the participants with an existing and validated measure. It is aimed at children between 5 and 14 years of age. It consisted of 19 items (raw scores 0–19) that evaluate a child’s use of language in concrete communicative situations and social interaction with respect to different speech acts such as greetings and farewells, requesting, giving or refusing permission, asking for specific information making questions (e.g., who/what, where/when, and why/how), or making direct demands for action, among others.

The child is presented with a black-and-white graphic scene (a vet’s clinic) with different characters. The child is the main character of the scene, and he is asked to produce a sentence that fits different interactions within the scene (e.g., the main character has to greet a woman he already knows in the clinic: “*what would he say to greet the woman?*”). In this sense, the child must understand the communicative intention of the character and say what the character should say in that particular situation (in the first person).

#### 2.4.3 Structural language formal measures

##### 2.4.3.1 Receptive grammar: CEG

The test *Comprensión de Estructuras Gramaticales* (CEG, 81) is a formal measure of grammatical comprehension for children aged 4 to 11 years, and it is the Spanish adaptation of the Test for Reception of Grammar (TROG) (82). This test allows the assessment of children’s ability to understand different types of grammatical structures that vary in length and degrees of complexity. The child hears a sentence and he must choose which of the four given pictures corresponds to it. It contains 80 items (raw score 0–80). The test has adequate psychometric properties: The internal consistency used as a measure of reliability (Cronbach’s alpha) showed an index of 0.91; Validity of criteria, correlation values: CEG-Peabody Picture Vocabulary Test (r = 0.809, *p* < 0.001) and CEG-Illinois Test Psycholinguistic Abilities (r = 0.644, *p* < 0.001; Peabody Picture Vocabulary Test) (83).

##### 2.4.3.2 Receptive vocabulary: PPVT-III

The Spanish version of the PPVT-III was used to assess the level of comprehensive vocabulary of the sample (83). It is aimed at children between 2 and 6 and 90 years old. The test items are organized in blocks of 12, each ordered by age. The total score can range between 0 and 192 points. This test has adequate psychometric properties: (a) Internal consistency of items: High reliabilities (minimum of 0.90) were reported for the 25 age groups of the norm sample with median reliability of 0.95; (b) Split half reliability: reliabilities ranged from 0.86 to 0.97 for the standardization age groups for both forms; and (c) Test–retest: corrected coefficients were reported between 0.91 to 0.94 with no difference in magnitude between the two forms (84).

#### 2.4.4 Theory of mind formal measure: TEC

The Test of Emotion Comprehension is a formal measure of emotional understanding for children between 3 and 11 years of age (85), and it was used to obtain a social cognition measure of the children. In this sense, the ability to understand emotions is linked with social cognition because understanding others’ emotions requires understanding the significance of the relations of other people with their goals and context (86). The Spanish version of the test is currently in the validation phase. Thus, to conduct the present study, one of the authors of the TEC provided the authors with the Spanish version of the instructions adapted by professors Carlos Hernández Blasi and Francisco Pons.

This instrument allows the formal assessment of nine components of emotion understanding: recognition of emotions (component 1), external causes (component 2), emotions based on desires (component 3), emotions based on beliefs (component 4), emotions based on memories (component 5), regulation of emotions (component 6), hiding emotions (component 7), mixed emotions (component 8), and moral emotions (component 9). The TEC raw score ranged from 0 to 9, and it was obtained by adding the sub-scores for the nine components. The TEC consists of 23 cartoon scenario stories (black-and-white pictures), and it is available in both girl and boy versions. A brief story is read by the examiner first, and then the child is asked to choose the correct facial expression (emotion) for the main character from among four given options of a combination of *happy*, *sad*, *angry*, *scared,* and/or *well*. As remarked by Fidalgo, Tenenbaum, and Aznar (87), the TEC has good test–retest reliability after a 3-month delay [*r*(18) = 0.84] and a good test–retest correlation after a 13-month delay [*r*(40) = 0.64 and *r*(32) = 0.54] (88).

#### 2.4.5 Non-verbal reasoning formal measure: Raven’s progressive matrices

The *Raven’s Progressive Matrices test* was administered to have a non-verbal reasoning score for each participant (RPM) (89). It is a multiple-choice visual task of abstract reasoning. The test requires the participant to infer a rule to generate the next items in a series or to determine whether a presented design is consistent with the rule. Items become progressively more difficult, building upon knowledge accumulated from the test. Nevertheless, as the age of the sample ranged from 5 to 12 years old, both General and Colored versions of the test were used, depending on the age of the participant.

In this sense, the *Colored Progressive Matrices test (CPM)* was aimed at children from 4 to 9 years of age; therefore, it was used to assess non-verbal reasoning and learning potential from participants in our sample aged between 5 and 9 years. It contains 36 items, so raw scores range between 0 and 36 points. The *Standard Progressive Matrices (SPM)* is aimed at people from 9 to 70 years of age, and it has 48 items, so raw scores range between 0 and 48 points. Hence, it was used to assess non-verbal reasoning and learning potential from participants in our sample from 10 to 12 years of age.

The standardization study generated a value of 0.80 in test–retest reliability (90).

### 2.5 Data analysis

First, the psychometric properties of PleaseApp are presented. For this purpose, confirmatory factor analysis (CFA), reliability analysis, and external (convergent) validity tests were performed. The data were found to follow a normal distribution (Z = 0.072; *p* = 0.053).

To empirically examine the factor structure of the test, a confirmatory factor analysis (CFA) based on structural equation programming (91, 92) was carried out using the AMOS 29 program with a sample of 153 participants. The variances of the latent variables were set at 1.0. The variances of the error terms were specified as free parameters. The maximum likelihood (ML) estimation method was used.

The following fit statistics were performed: the Satorra-Bentler chi-square (ꭓ^2^), the standardized chi-square (ꭓ2/df), the statistical likelihood (p), the root mean square error of approximation (RMSEA), the comparative fit index (CFI), the Tucker–Lewis index (TLI), and the standardized root mean square residual (SRMR). The reliability of the questionnaire is examined using Cronbach’s alpha coefficient (93), with factor loadings obtained in the CFA and corrected correlations. Descriptive analyses of each item, such as mean, standard deviation, skewness, and kurtosis, are also calculated. To analyze the correlation among the different levels and the relationship between PleaseApp levels and other related measures (convergent validity), Pearson’s correlation is used.

Second, an analysis of the data obtained is presented. For this purpose, to determine the level of the children in each dimension measured, as well as the differences between the variables sex and age, descriptive analyses (mean and standard deviation) and statistical analyses were performed using Student’s *t*-test and analysis of variance (ANOVA) with the corresponding size effects (Cohen’s d and Eta squared). Taking into account the ANOVA results, Tukey’s post-hoc is calculated.

## 3 Results

### 3.1 Psychometric properties

#### 3.1.1 Confirmatory factor analysis

A first model of eight first-order factors and one second-order factor was tested. In this sense, PleaseApp, as a test of pragmatics and social communication, was positioned as a second-order factor (exogenous latent variable), and the test of narrative (0.70), politeness (0.84), reference (0.57), indirect speech acts (0.80), complex intentionality (0.81), gesture-speech integration (0.52), humor (0.10), and figurative language (0.58), were positioned as factors of first order (endogenous latent variables). This Model 1 did not yield acceptable fits (ꭓ^2^ = 331.210, gl = 197, *p* < 0.000; ꭓ2/gl = 1.68; CFI = 0.721; TLI = 0.735; RMSEA = 0.071, SRMR = 0.074).

Subsequently, a second first-order model (Model 2) was tested with eight factors related to each other, forming a network of interrelationships. Although the covariances between the factors ranged between values of 0.32 and 0.78, this model did not yield acceptable fits (*ꭓ2* = 421.12, *gl* = 198, *p* < 0.000; ꭓ2/gl = 2.12; CFI = 0.713; TLI = 0.698; RMSEA = 0.073, SRMR = 0.078).

Finally, the structure that showed better properties was an eight-factor one without an underlying structure for these factors. Table 3 displays the model of each factor.

**Table 3:** Values of the indices used to evaluate the fit of the models of the eight factors that make up PleaseApp.

	ꭓ^2^	df	*p*	ꭓ^2^/df	CFI	TLI	RMSEA	SRMR
Narrative (cinema)	78.979	42	0.000	1.88	0.923	0.901	0.040	0.0583
Politeness (train)	62.857	39	0.009	1.61	0.931	0.903	0.032	0.0625
Reference (kitchen)	36.499	17	0.004	2.14	0.980	0.967	0.048	0.0355
Indirect speech acts (school)	14.720	13	0.032	1.13	0.971	0.953	0.000	0.0456
Complex intentionality (beach)	30.335	17	0.024	1.78	0.900	0.935	0.026	0.0642
Gesture-speech integration (circus)	19.230	18	0.037	1.06	0.990	0.984	0.000	0.0520
Humor (TV)	33.750	20	0.028	1.68	0.952	0.933	0.023	0.0563
Understanding of figurative language (zoo)	75.677	43	0.002	1.75	0.933	0.914	0.044	0.0607

χ2, chi-square; df, degrees of freedom; p, overall model significance; ꭓ2/df, normalized chi-square; CFI, comparative fit index; TLI, Tucker–Lewis index; RMSEA, mean square error of approximation; SRMR, standardized root mean square residual.

As can be seen in Table 3, all the indices are in the desirable ranges, indicating a good fit of the models.

**Figure 2:**
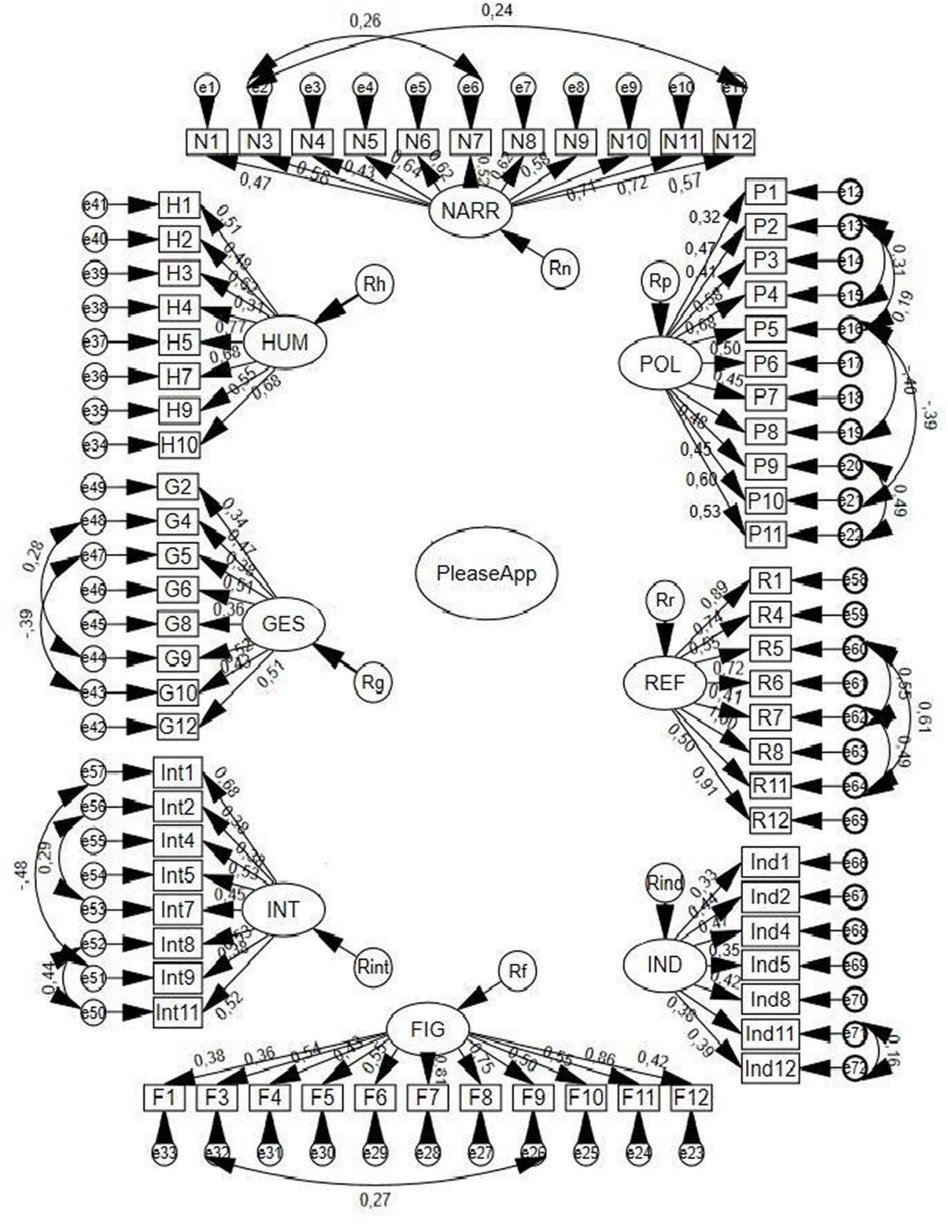
Diagram of the 8 models that compose PleaseApp with standardized parameter values.

The estimated values of the parameters are presented graphically in Figure 2, all being statistically significant.

To find out the relationship between the different levels measured, Table 4 shows the correlations between each of the levels with the total PleaseApp.

**Table 4:** Correlations (Pearson’s r) between the eight levels with each other and with PleaseApp.

	NARR	POL	REF	GES	IND	INT	HUM	FIG
POL	0.327^**^							
REF	0.073	0.040						
GES	0.307^**^	0.068	0.091					
IND	0.248^**^	0.311^**^	−0.080	0.207^*^				
INT	0.259^**^	0.297^**^	0.112	0.312^**^	0.341^**^			
HUM	0.123	0.005	0.170^*^	0.135	−0.099	0.039		
FIG	0.469^**^	0.464^*^	0.015	0.344^**^	0.289^**^	0.468^**^	0.063	
PleaseApp	0.725^**^	0.545^**^	0.203^*^	0.550^**^	0.462^**^	0.593^**^	0.409	0.762^**^

^**^p < 0.01; ^*^p < 0.05; NARR, narrative (cinema); POL, politeness (train); REF, reference (kitchen); IND, indirect speech acts (school); INT, complex intentionality (beach); GES, gesture-speech integration (circus); HUM, humor (TV); FIG, figurative language (zoo).

As can be seen, *narrative ability (NARR)* is significantly related to all dimensions except reference skills and humor. The dimension of *gesture-speech integration (GES)* correlates with the understanding of *indirect speech acts (IND)*, *complex intentionality (INT)*, and figurative language (FIG). *Referencing ability (REF)* is significantly related to the understanding of *verbal and visual humor (HUM)*. Comprehension *of indirect speech acts (IND)* is related to complex intentionality (INT), politeness (POL), and understanding of figurative language (FIG). *Complex intentionality (INT)* is correlated with politeness (POL) and understanding of figurative language (FIG); and *politeness* is also related to the understanding of figurative language (FIG). Furthermore, as shown in the Table, all dimensions, except humor, are related to the total dimension of PleaseApp.

#### 3.1.2 Reliability

The reliability of the internal consistency of the scores of the PleaseApp levels and the items that compose them is estimated with Cronbach’s alpha (α).

The values of the internal consistency alpha coefficient for each level are narrative (cinema level; α = 0.795); politeness (train level; α = 0.753); reference (cooking level; α = 0.792); indirect speech acts (school level; α = 0.776); complex intentionality (beach level; α = 0.704); gesture-speech integration (circus level; α = 0.710); humor (TV level; α = 0.773); and figurative language (zoo level; α = 0.819).

As can be seen, all levels obtain an adequate alpha value, between 0.704 and 0.81, so that all levels have a satisfactory internal consistency.

Next, for each of the levels, the mean score, standard deviation, skewness, and kurtosis are shown below. The corrected correlations and the reliability of each test are presented using Cronbach’s alpha coefficient. Similarly, changes in Cronbach’s alpha are estimated if any item is eliminated (see Table 5).

**Table 5:** Descriptive statistics of the items and item-total correlation of each of the test.

Items NARR	M (SD) 0–2	Skewness	Kurtosis	Corrected correlation	α if deleted
NARR 3	1.50 (0.765)	−0.982	0.780	0.406	0.785
NARR 4	1.40 (0.896)	−0.969	−0.042	0.463	0.780
NARR 5	1.75 (0.602)	−0.865	−0.807	0.521	0.772
NARR 6	1.66 (0.722)	−0.717	0.970	0.455	0.778
NARR 7	1.68 (0.687)	−0.865	0.710	0.443	0.779
NARR 8	1.67 (0.690)	−0.824	0.635	0.418	0.782
NARR 9	1.48 (0.847)	−0.700	0.156	0.483	0.776
NARR 10	1.89 (0.392)	−0.865	−0.561	0.479	0.782
NARR 11	1.71 (0.659)	−0.300	0.021	0.601	0.762
NARR 12	1.25 (0.757)	−0.860	0.750	0.508	0.772
**Items POL**	**M (SD) 0–2**	**Skewness**	**Kurtosis**	**Corrected correlation**	**α if deleted**
POL 2	1.89 (0.438)	−0.293	0.282	0.346	0.741
POL 3	1.91 (0.362)	−0.827	0.686	0.445	0.728
POL 4	1.93 (0.318)	−0.499	0.831	0.430	0.727
POL 5	1.92 (0.354)	−0.544	0.018	0.447	0.727
POL 6	1.85 (0.456)	−0.501	0.944	0.461	0.726
POL 7	1.88 (0.434)	−0.981	0.863	0.407	0.717
POL 8	1.90 (0.426)	−0.588	0.715	0.517	0.701
POL 9	1.94 (0.286)	−0.332	0.758	0.340	0.727
POL 10	1.83 (0.456)	−0.544	0.918	0.278	0.736
POL 11	1.89 (0.383)	−0.628	0.679	0.494	0.706
**Items REF**	**M (SD) 0–2**	**Skewness**	**Kurtosis**	**Corrected correlation**	**α if deleted**
REF 1	1.99 (0.114)	−0.889	0.966	0.717	0.775
REF 4	1.99 (0.162)	−0.824	0.771	0.658	0.782
REF 5	1.78 (0.413)	−0.028	0.953	0.719	0.774
REF 6	1.96 (0.226)	−0.818	0.876	0.634	0.782
REF 7	1.78 (0.417)	−0.645	0.710	0.581	0.796
REF 8	1.76 (0.426)	−0.792	0.856	0.835	0.770
REF 11	1.99 (0.114)	−0.478	0.699	0.651	0.782
REF 12	1.99 (0.114)	−0.605	0.961	0.801	0.770
**Items IND**	**M (SD) 0–2**	**Skewness**	**Kurtosis**	**Corrected correlation**	**α if deleted**
IND 2	1.55 (0.777)	−0.893	0.540	0.338	0.721
IND 4	1.69 (0.642)	−1.172	0.455	0.336	0.723
IND 5	1.65 (0.748)	−1.134	0.843	0.317	0.771
IND 8	1.70 (0.660)	−0.651	0.838	0.314	0.746
IND 11	1.71 (0.646)	−0.210	0.587	0.397	0.738
IND 12	1.76 (0.596)	−1.191	0.907	0.395	0.740
**Items INT**	**M (SD) 0–2**	**Skewness**	**Kurtosis**	**Corrected correlation**	**α if deleted**
INT 1	1.97 (0.241)	−0.727	0.953	0.407	0.677
INT 2	1.82 (0.465)	−0.549	0.768	0.413	0.672
INT 4	1.80 (0.566)	−0.534	0.538	0.360	0.683
INT 5	1.77 (0.556)	−0.618	0.697	0.400	0.674
INT 7	1.82 (0.555)	−0.734	0.998	0.424	0.668
INT 8	1.90 (0.400)	−0.859	0.560	0.332	0.688
INT 9	1.69 (0.702)	−0.973	0.521	0.429	0.671
INT 11	1.86 (0.465)	−0.300	0.856	0.429	0.668
**Items GES**	**M (SD) 0–2**	**Skewness**	**Kurtosis**	**Corrected correlation**	**α if deleted**
GES 2	1.97 (0.178)	−0.342	0.781	0.309	0.678
GES 4	1.73 (0.553)	−0.233	0.365	0.388	0.670
GES 5	1.95 (0.251)	−0.901	0.295	0.344	0.623
GES 6	1.59 (0.683)	−0.605	0.196	0.420	0.656
GES 7	1.27 (0.941)	−0.635	−0.558	0.334	0.696
GES 9	1.90 (0.447)	−0.416	0.319	0.406	0.673
GES 10	1.12 (0.962)	−0.257	−0.875	0.380	0.679
GES 12	1.78 (0.549)	−0.577	0.365	0.385	0.671
GES 12	1.78 (0.549)	−0.577	0.365	0.385	0.671
**Items HUM**	**M (SD) 0–2**	**Skewness**	**Kurtosis**	**Corrected correlation**	**α if deleted**
HUM 1	1.39 (0.897)	−0.778	−0.316	0.422	0.759
HUM 2	1.73 (0.651)	−0.968	0.165	0.460	0.752
HUM 3	1.35 (0.815)	−0.880	−0.820	0.503	0.743
HUM 4	1.43 (0.705)	−0.899	−0.456	0.183	0.790
HUM 5	1.64 (0.766)	−0.682	0.882	0.657	0.717
HUM 7	1.32 (0.856)	−0.714	−0.214	0.585	0.728
HUM 9	1.25 (0.905)	−0.526	−0.605	0.561	0.732
HUM 10	1.25 (0.763)	−0.529	−0.124	0.422	0.755
**Items FIG**	**M (SD) 0–2**	**Skewness**	**Kurtosis**	**Corrected correlation**	**α if deleted**
Fig 2	1.43 (0.696)	−0.729	−0.486	0.329	0.816
Fig 3	1.80 (0.501)	−0.474	0.384	0.452	0.804
Fig 4	1.70 (0.514)	−0.489	0.263	0.503	0.799
Fig 5	1.74 (0.559)	−0.205	0.991	0.382	0.810
Fig 6	1.65 (0.633)	−0.723	0.739	0.507	0.798
Fig 7	1.63 (0.498)	−0.89	−0.763	0.711	0.782
Fig 8	1.69 (0.493)	−0.236	0.469	0.615	0.791
Fig 9	1.66 (0.598)	−0.738	0.104	0.413	0.807
Fig 10	1.52 (0.699)	−0.194	0.084	0.485	0.801
Fig 11	1.70 (0.460)	−0.953	−0.107	0.752	0.781
Fig 12	1.45 (0.716)	−0.199	−0.059	0.362	0.816

NARR, narrative (cinema); POL, politeness (train); REF, reference (kitchen); IND, indirect speech acts (school); INT, complex intentionality (beach); GES, gesture-speech integration (circus); HUM, humor (TV); FIG, figurative language (zoo).

As can be seen, all the correlation values between the item and the corrected total (eliminating the item, corrected homogeneity index) exceed the value of 0.30, and there is no improvement in the Cronbach’s alpha value if any item is eliminated; therefore, we cannot distinguish any weak item in the test.

#### 3.1.3 Convergent validity

To analyze the content validity of the different levels of PleaseApp, the participant sample was administered other instruments that assess factors associated with the development of pragmatics, such as non-verbal reasoning, structural language (grammar and vocabulary), and theory of mind (different components of emotion comprehension). Moreover, an existing pragmatic subtest of a Spanish Language Battery was also administered as a direct similar measure test of pragmatics. The correlations between these tests and the PleaseApp levels are presented in Table 6.

**Table 6:** Correlations (Pearson’s r) between PleaseApp levels and PleaseApp total score, with direct measures of non-verbal reasoning, vocabulary, grammar, theory of mind, and pragmatics.

	BLOC (pragmatics)	CEG	PPVT-III	TEC	Raven (CPM) *n* = 101	Raven (SPM) *n* = 52
NARR	0.397^**^	0.542^**^	0.603^**^	0.376^**^	0.552^**^	0.464^**^
POL	0.486^**^	0.593^**^	0.513^**^	0.205^**^	0.374^**^	−0.104
REF	−0.008	0.029	−0.034	0.114	−0.005	0.227
IND	0.274^**^	0.296^**^	0.275^**^	0.164^*^	0.312^**^	−0.060
INT	0.216^**^	0.371^**^	0.330^**^	0.259^**^	0.328^**^	0.197
GES	0.107	0.269^**^	0.305^**^	0.160^*^	0.165	0.402^**^
HUM	0.024	0.051	0.089	0.074	−0.019	0.377^**^
FIG	0.322^**^	0.605^**^	0.668^**^	0.376^**^	0.566^**^	0.416^**^
PleaseApp	0.444^**^	0.655^**^	0.686^**^	0.427^**^	0.607^**^	0.529^**^

NARR, narrative (cinema); POL, politeness (train); REF, reference (kitchen); IND, indirect speech acts (school); INT, complex intentionality (beach); GES, gesture-speech integration (circus); HUM, humor (TV); FIG, figurative language (zoo); BLOC, Batería de lenguaje objetiva y criterial; CEG, Comprensión de Estructuras Gramaticales (receptive grammar); PPVT-III, Peabody (receptive vocabulary); TEC, Test of Emotion Comprehension (theory of mind) CPM, Raven’s Colored Progressive Matrices test (non-verbal reasoning); SPM, Raven’s Standard Progressive Matrices (non-verbal reasoning).

First, in relation to the non-verbal reasoning measure, a positive and strong correlation of the scores was observed between the participant’s performance in general (PleaseApp) and the scores in both CPM and SPM. Table 6 also shows the specific correlations of each level with this non-verbal measure. In this sense, the data in the first two columns indicate that performance on all levels, except gesture-speech integration (GES), was correlated with participants’ non-verbal reasoning skills.

Second, in relation to structural language measures, a positive and strong correlation of the scores obtained by the participants in the tool in general (PleaseApp) was observed with both the grammatical measure (CEG) and the vocabulary measure (Peabody). Table 6 also shows the specific correlations of each level with both receptive measures of structural language. In this regard, the data in the third and fourth columns indicate that all levels, with the exception of reference (REF) and humor (HUM), were correlated with participants’ structural language skills.

Similarly, a positive and strong correlation of the scores obtained by the participants in the tool in general (PleaseApp) was also observed with the theory of mind measure (TEC). Table 6 also shows the specific correlations of each level with the theory of mind measure. In this regard, the data in the fifth column indicate that all levels, with the exception of reference (REF) and humor (HUM), were correlated with participants’ theory of mind skills.

Finally, a strong positive correlation between the scores obtained by the participants on the tool in general (PleaseApp) and the pragmatic measure of BLOC was observed. Table 6 also shows the specific correlations of each level with the BLOC expressive pragmatics measure. In this regard, the data in the last column indicate that all levels, with the exception of gesture-speech integration (GES), reference (REF), and humor (HUM), were correlated with participants’ expressive pragmatic skills.

### 3.2 Analysis of demographic differences (age and sex)

To analyze the differences in the different levels by sex, a comparison of means for independent samples was carried out. The data are presented in Table 7.

**Table 7:** Differences by sex of the participants in the different levels.

	Boys	Girls	*p*	*d*
	M (DT)	M (DT)		
NARR (0–20)	15.92 (4.51)	16.06 (3.89)	0.418	−0.034
POL (0–20)	18.73 (2.36)	19.19 (2.01)	0.104	−0.207
REF (0–16)	15.04 (1.08)	15.51 (1.02)	0.003	−0.447
IND (0–12)	9.81 (2.37)	10.36 (1.79)	0.056	−0.295
INT (0–16)	14.43 (2.16)	14.83 (2.05)	0.125	−0.188
GES (0–16)	13.09 (2.45)	13.67 (2.41)	0.068	−0.245
HUM (0–16)	11.48 (3.81)	11.20 (4.17)	0.337	0.069
Fig (0–22)	17.87 (3.81)	18.07 (3.85)	0.372	−0.053
PleaseApp (0–138)	116.37 (13.1)	118.82 (12.0)	0.120	−0.194

NARR, narrative ability (cinema); POL, politeness formulas (train); REF, reference skills (cooking); IND, indirect language comprehension (school); INT, complex communicative intentionality (beach); GES, gesture-speech integration (circus); HUM, visual and verbal humor comprehension (TV); FIG, understanding of figurative language (zoo).

As can be seen, in general, no significant differences were found between boys and girls. Specifically, significant differences were found in the reference level, although the small effect size should be taken into account (*d* = −0.44).

Regarding the age of the children, Table 8 shows the means and standard deviations by the eight age ranges, both in the general measure PleaseApp and in the different levels.

**Table 8:** Means and standard deviation in PleaseApp and each level by age group.

Age	NARR (0–20)	POL (0–20)	REF (0–16)	IND (0–12)	INT (0–16)	GES (0–16)	HUM (0–16)	FIG (0–22)	PleaseApp (0–138)
5 (n = 13)	10 (6.11)	16.62 (2.5)	15.31 (0.94)	8 (1.73)	13.62 (1.85)	13 (1.82)	11.77 (2.35)	14.62 (2.75)	102.92 (7.72)
6 (n = 16)	11.88 (5.43)	17.06 (3.1)	15.44 (1.09)	9.75 (1.91)	14.0 (2.09)	12.63 (2.41)	11.38 (3.0)	13.88 (2.06)	106 (12.61)
7 (n = 24)	15.48 (3.1)	18.46 (3.45)	15.33 (0.917)	10 (2.37)	13.83 (2.69)	12.79 (3.21)	12.63 (2.10)	15.96 (3.60)	114.08 (11.15)
8 (n = 19)	16.05 (3.18)	19.33 (1.08)	15.47 (0.90)	9.53 (2.45)	15.37 (1.06)	13.00 (2.22)	11.05 (4.07)	17.89 (3.51)	117.88 (9.86)
9 (n = 20)	17.25 (2.42)	19.40 (1.04)	15.50 (0.889)	10.05 (1.90)	13.75 (3.33)	12.10 (2.59)	11.85 (3.80)	19.05 (3.74)	118.95 (12.31)
10 (n = 24)	18.09 (1.99)	19.91 (0.47)	14.96 (1.97)	10.83 (2.03)	15.08 (1.28)	14.35 (1.94)	9.54 (5.47)	20.54 (2.16)	123.39 (8.33)
11 (n = 26)	18.23 (1.92)	19.88 (0.32)	15 (1.29)	10.77 (1.65)	15.65 (0.93)	14.15 (2.18)	11.35 (4.34)	19.81 (3.13)	125.03 (9.92)
12 (n = 11)	17.91 (2.42)	19.73 (0.64)	15.09 (1.3)	10.64 (2.06)	15.09 (1.44)	14.55 (1.36)	11.64 (4.82)	20.36 (2.80)	125 (11.77)

NARR, narrative (cinema); POL, politeness (train); REF, reference (kitchen); IND, indirect speech acts (school); INT, complex intentionality (beach); GES, gesture-speech integration (circus); HUM, humor (TV); FIG, figurative language (zoo).

Thus, to know if there were differences in the scores obtained according to the age of the participants, an ANOVA was carried out. The data indicate that there were differences according to the age of the participants in six out of eight levels (NARR: *F* = 13.06, *p* < 0.001, *η^2^ = 0.*39; GES: *F* = 3.01, *p* = 0.006, *η^2^ = 0.12;* IND: *F* = 3.19, *p* = 0.004, *η^2^ = 0.*13; INT: *F* = 3.36, *p* = 0.002, *η*^2^ = 0.14; POL: *F* = 7.155, *p* < 0.001, *η*^2^ = 0.25; FIG: *F* = 12.72, *p* < 0.001, *η*^2^ = 0.33), except in the reference (REF: *F* = 0.845, *p* = 0.552, *η*^2^ = 0.03) and humor levels (HUM: *F* = 1.162, *p* = 0.329, *η*^2^ = 0.05). Similarly, significant differences by age were found in the total measure PleaseApp (*F* = 10.413, *p* < 0.001, η^2^ = 0.33). Table 9 presents the means and standard deviations by age groups. In this sense, to find out whether there were significant differences in means between the different age groups in the different levels measured in PleaseApp, a post-hoc analysis (Tukey) was conducted. Results showed that, for each level, there is a significant jump at different ages.

**Table 9:** Differences between each of the ages at all levels.

Age		NARR	POL	REF	IND	INT	GES	HUM	FIG	PleaseApp
		*p*	*p*	*p*	*p*	*p*	*p*	*p*	*p*	
5	6	0.815	0.999	1.000	0.304	1.000	1.000	1.000	0.998	0.990
	7	<0.001	0.119	1.000	0.093	1.000	1.000	0.998	0.910	0.052
	8	<0.001	0.005	1.000	0.436	0.240	1.000	1.000	0.069	0.005
	9	<0.001	0.002	1.000	0.099	1.000	0.759	1.000	0.002	0.001
	10	<0.001	<0.001	0.982	0.002	0.408	0.880	0.729	<0.001	<0.001
	11	<0.001	<0.001	0.991	0.003	0.065	0.952	1.000	<0.001	<0.001
	12	<0.001	0.004	1.000	0.041	0.628	0.875	1.000	<0.001	<0.001
6	7	0.029	0.349	1.000	1.000	1.000	1.000	0.977	0.422	0.308
	8	0.009	0.020	1.000	1.000	0.483	1.000	1.000	0.004	0.042
	9	<0.001	0.011	1.000	1.000	1.000	0.794	1.000	<0.001	0.013
	10	<0.001	<0.001	0.868	0.723	0.708	0.749	0.839	<0.001	<0.001
	11	<0.001	<0.001	0.907	0.767	0.169	0.871	1.000	<0.001	<0.001
	12	<0.001	0.015	0.992	0.954	0.863	0.775	1.000	<0.001	<0.001
7	8	0.999	0.839	1.000	0.995	0.211	1.000	0.900	0.454	0.964
	9	0.679	0.754	1.000	1.000	1.000	0.822	0.998	0.025	0.845
	10	0.159	0.182	0.930	0.850	0.387	0.479	0.131	<0.001	0.074
	11	0.093	0.172	0.958	0.886	0.036	0.645	0.946	<0.001	0.014
	12	0.513	0.632	0.999	0.989	0.677	0.584	0.997	0.003	0.109
8	9	0.955	1.000	1.000	0.993	0.199	0.920	0.998	0.939	1.000
	10	0.527	0.981	0.778	0.431	1.000	0.484	0.917	0.103	0.694
	11	0.399	0.984	0.832	0.475	1.000	0.641	1.000	0.447	0.366
	12	0.833	0.999	0.982	0.840	1.000	0.568	1.000	0.410	0.627
9	10	0.992	0.989	0.717	0.910	0.365	0.021	0.535	0.750	0.854
	11	0.977	0.991	0.776	0.935	0.037	0.038	1.000	0.991	0.545
	12	1.000	1.000	0.973	0.995	0.638	0.064	1.000	0.948	0.776
10	11	1.000	1.000	1.000	1.000	0.974	1.000	0.743	0.990	1.000
	12	1.000	1.000	1.000	1.000	1.000	1.000	0.830	1.000	1.000
11	12	1.000	1.000	1.000	1.000	0.994	1.000	1.000	1.000	1.000

NARR, narrative (cinema); POL, politeness (train); REF, reference (kitchen); IND, indirect speech acts (school); INT, complex intentionality (beach); GES, gesture-speech integration (circus); HUM, humor (TV); FIG, figurative language (zoo).

## 4 Discussion

The aim of the present study was to evaluate the psychometric characteristics of the digital tool PleaseApp as a formal assessment of receptive pragmatics in a sample of primary school children and to analyze the data obtained, taking into account the age and sex of the participants.

The field of study of pragmatics has been supported by different professional disciplines and theoretical approaches, as well as the clinical implications for the assessment of pragmatic skills in different populations with NDD. This fact motivated the authors to develop PleaseApp as a digital tool for the formal assessment of receptive pragmatic skills.

The development of PleaseApp included different phases. In the first phase, the different pragmatic components were decided based on previous scientific evidence. For this purpose, on the one hand, several theoretical and empirical studies on the developmental milestones of pragmatic skills in typically developing children were reviewed, as well as the scientific procedures for the assessment of these skills. On the other hand, we also reviewed the specific needs detected in populations with different NDD that have primary language and/or communication difficulties, above all those coming from ASD and DLD samples and also those coming from other disorders who also suffer secondary pragmatic impairments such as children with ADHD.

PleaseApp is composed of eight levels, with item variation within each level: (1) narrative (cinema level), which assesses the child’s ability to order sequences of a story (mechanical, behavioral, and mentalistic); (2) politeness (train level), which assesses the ability to identify correct politeness formulas in a specific situation; (3) reference (kitchen level), which assesses the understanding of the optimality of reference expressions and when an expression is under informative); (4) indirect speech acts (school level), which aims to assess the ability to detect indirect communicative intentions and hints; (5) complex intentionality (beach level), which assesses the understanding of masked communicative intentionality, specifically the detection of intentionality/non-intentionality, the use of irony (sarcasm, questions, assertions, and exaggerations), and the valence of the intention (positive/negative); (6) gesture-speech integration (circus level), which assesses the ability to understand meanings when a speaker integrates iconic gestures with speech; (7) humor (TV level), which assesses comprehension of humor, specifically the detection of incongruities (visual/verbal), and type of humor (puns, semantic analogy, and jokes that include theory of mind); and (8) figurative language (zoo level), which assesses comprehension of non-literal language, specifically metaphors (novel and conventional) and similes.

After testing the structure of two models, one with eight first-order factors and one second-order factor and a second model with eight interrelated factors, these did not yield acceptable fits. Therefore, an eight-factor model without an underlying structure to these factors was the one that showed better properties. The eight tests that makeup PleaseApp have obtained a model with adequate adjustments and with adequate reliability and validity indexes, so it is considered an adequate evaluation instrument to measure these eight aspects.

The significant correlations between all these dimensions with the variable PleaseApp (total score), except for humor, indicated the existence of an association between the components assessed with pragmatics. Nevertheless, in relation to the humor component, there may be different explanations for why it was not correlated with the total score of PleaseApp. First, this component includes both visual and verbal items to grasp the funny meaning, and perhaps only verbal ones are more related to pragmatic competence but not the total visual ones. Second, it is observed that from all the related measures, it only correlates with non-verbal reasoning (and in children from 9 to 12 years old, since this correlation is only observed in the group in which the *Standard Progressive Matrices* have been used). Third, as happens in the reference component, age does not contribute to differentiating the abilities of the sample since the means are very similar in the different age groups assessed. In this sense, it seems that, on the one hand, humor is a skill that is more related to the ability to find creative solutions to a problem (especially from a visual level), which relies more on the pragmatic skills of the subject when the joke includes verbal information. Future studies on each specific component should study this issue in depth, considering both item variation of the component and its association with different cognitive, linguistic, and social aspects related to pragmatics.

Similarly, significant correlations were observed between the PleaseApp components with non-verbal reasoning, structural language (i.e., grammar and vocabulary), and theory of mind as related factors associated with the development of pragmatics. Moreover, a significant correlation was observed between PleaseApp and an independent pragmatic standardized formal measure. Therefore, the relationship between the aspects evaluated in PleaseApp and pragmatics abilities measured externally is confirmed. However, it must be noted that the TEC measure was used as a formal measure of theory of mind and that this measure tests nine different components of emotional understanding (including complex aspects such as morality), and its raw score was obtained by adding the sub-scores (ranging from 0 to 9). In this sense, although it is a good measure that covers different periods of development of the affective theory of mind skills, the range of scores that it offers is not as wide as the other instruments used in the present study to offer a complete profile of social cognition of the participants. So, future studies should study the relationship of the pragmatic subcomponents of PleaseApp with more measures of social cognition to map the whole competence (94) to better conceptualize existing impairments in the different neurodevelopmental conditions (95, 96).

Except for the reference level, no significant differences were found taking into account the sex of the participants, although it should be noted that girls obtained higher mean levels in the different dimensions. In contrast, there are significant differences according to the age of the participants in all levels except for reference level (kitchen) and humor level (TV). In this regard, means of the different age groups showed a progressive increase in all the levels.

One of the main contributions of PleaseApp to the research and clinical community is the provision of an evidence-based, comprehensive approach to multiple skills linked to pragmatic development in children from 5 to 12 years of age. It allows the assessment of pragmatic ability in eight areas with direct measurements, determining a general idea of age-appropriate pragmatic skills of the child in comparison to the sample of reference, and also regarding the strengths and weaknesses in each component. Moreover, it is possible to find out relevant aspects related to each component that will allow us to disentangle different difficulties in different disorders (e.g., processing theory of mind content in narrative skills and the rest of the areas assessed), as their variation of items according to the theoretical basis, and also is possible to find out what type of mistakes children are having when using their pragmatic skills (e.g., literal interpretation vs. incoherent interpretation). Coding participants’ responses for pragmatic appropriateness is important to investigate what type of inference the person is making, and to what extent they benefit from contextual cues or understanding of the interlocutor’s intention [e.g., (31, 48)]. In this sense, it is a tool that will help future studies assessing neurodivergent children to state strengths and difficulties in different aspects of pragmatics and to study in depth its association with different aspects of the disorder (primary or secondary). Finally, another contribution is that it is a receptive formal measure that will allow us to triangulate information with other ecological and formal expressive instruments available in our context [e.g., CCC-2) (63); CORP (97); or CELF-5 (64)].

PleaseApp has also been designed to motivate children when they are being assessed. In this sense, the different levels have been contextualized in familiar environments and activities for children to provide a functional and meaningful environment for assessment. Moreover, PleaseApp is a digital tool created digitally from its origin, that is, it is not an adaptation of an existing tool, although it is based on the study of different tasks and manuals, books, and existing tests of pragmatics for the theoretical preparation of its structure and its items. In this sense, it facilitates the assessment and the correction by professionals, and its digital features make it appropriate for the assessment of both typically developing children and children with NDDs, as has been demonstrated in other studies (62, 63), above all to compensate for cognitive and linguistic aspects when being assessed such as oral expression, language comprehension, attention, speed processing, imagination, or working memory, among others.

Therefore, PleaseApp is an easy tool to use based on scientific background, and it has adequate psychometric properties to be implemented in different contexts, such as clinical health, social health, and education. Future research could address the integration of other pragmatic skills, such as metapragmatics in conversation, new lexical contextual inference ability, or quantifier comprehension.

Furthermore, the success of the communicative situation or dialog not only depends on the pragmatic receptive skills as a listener but also on the expressive skills of the speaker (25, 58). In this sense, expressive formal measures would complete a picture of the actual strengths and difficulties a child has in the area of pragmatics. In this sense, although PleaseApp allows to offer communicative contexts with multimodal details as similar as possible to real situations, it is not as ecological as real communicative contexts, and a professional must match the information obtained with other pragmatic ecological assessments (e.g., observational measures, interviews to caregivers, or questionnaires) to have a better picture of the actual strengths and difficulties of pragmatic behaviors of children in real interactions with people.

As a general conclusion, the importance of the present results for research is also highlighted because this is the first empirical study that measures different pragmatic components in the same typically developing sample of 5- to 12-year-olds. In addition, evidence is provided that the PleaseApp has adequate psychometric properties and, therefore, can be used reliably and validly in the assessment of these pragmatic aspects in primary school children with NDD.

## Data availability statement

The datasets generated for this study are available from the corresponding author upon reasonable request. Requests to access the datasets should be directed to RF-B, flores@uji.es.

## Ethics statement

The studies involving humans were approved by Universitat Jaume I de Castellón. The studies were conducted in accordance with the local legislation and institutional requirements. Written informed consent for participation in this study was provided by the participants’ legal guardians/next of kin.

## Author contributions

CA-R: Conceptualization, Data curation, Formal analysis, Funding acquisition, Investigation, Methodology, Project administration, Resources, Software, Supervision, Validation, Visualization, Writing – original draft, Writing – review & editing. RF-B: Conceptualization, Data curation, Formal analysis, Funding acquisition, Investigation, Methodology, Project administration, Resources, Software, Supervision, Validation, Visualization, Writing – original draft, Writing – review & editing. AI: Conceptualization, Data curation, Formal analysis, Investigation, Methodology, Project administration, Resources, Software, Supervision, Validation, Visualization, Writing – original draft, Writing – review & editing.
